# To Self-Treat or Not to Self-Treat: Evaluating the Diagnostic, Advisory and Referral Effectiveness of ChatGPT Responses to the Most Common Musculoskeletal Disorders

**DOI:** 10.3390/diagnostics15141834

**Published:** 2025-07-21

**Authors:** Ufuk Arzu, Batuhan Gencer

**Affiliations:** Department of Orthopedics and Traumatology, Marmara University Pendik Training and Research Hospital, 34890 Istanbul, Turkey; gencer.batuhan@gmail.com

**Keywords:** ChatGPT, self-diagnosis, self-treatment, readability, Flesch–Kincaid Grade Level, trauma

## Abstract

**Background/Objectives**: The increased accessibility of information has resulted in a rise in patients trying to self-diagnose and opting for self-medication, either as a primary treatment or as a supplement to medical care. Our objective was to evaluate the reliability, comprehensibility, and readability of the responses provided by ChatGPT 4.0 when queried about the most prevalent orthopaedic problems, thus ascertaining the occurrence of misguidance and the necessity for an audit of the disseminated information. **Methods:** ChatGPT 4.0 was presented with 26 open-ended questions. The responses were evaluated by two observers using a Likert scale in the categories of diagnosis, recommendation, and referral. The scores from the responses were subjected to subgroup analysis according to the area of interest (AoI) and anatomical region. The readability and comprehensibility of the chatbot’s responses were analyzed using the Flesch–Kincaid Reading Ease Score (FRES) and Flesch–Kincaid Grade Level (FKGL). **Results:** The majority of the responses were rated as either ‘adequate’ or ‘excellent’. However, in the diagnosis category, a significant difference was found in the evaluation made according to the AoI (*p* = 0.007), which is attributed to trauma-related questions. No significant difference was identified in any other category. The mean FKGL score was 7.8 ± 1.267, and the mean FRES was 52.68 ± 8.6. The average estimated reading level required to understand the text was considered as “high school”. **Conclusions:** ChatGPT 4.0 facilitates the self-diagnosis and self-treatment tendencies of patients with musculoskeletal disorders. However, it is imperative for patients to have a robust understanding of the limitations of chatbot-generated advice, particularly in trauma-related conditions.

## 1. Introduction

The significance of herbal medicines and conventional treatment protocols such as RICE (rest, ice, compression, elevation) in the management of musculoskeletal disorders has resulted in an increase in patients’ attempts at self-diagnosis and self-treatment [1,2,3,4,5]. The advent of the internet and social media platforms, coupled with the increasing accessibility of artificial intelligence, has led to a notable enhancement in the ease with which information can be accessed on a daily basis [6,7,8]. The increased accessibility of information has resulted in a rise in patients trying to self-diagnose, conducting preliminary research on their diseases, and opting for self-medication, either as a primary treatment or as a supplement to medical care [5,6,9]. Furthermore, the global experience of the 2020 pandemic has contributed to a heightened awareness of the importance of self-care and a shift in attitudes towards medical visits [10,11,12]. The prevailing concern regarding infection in healthcare facilities, compounded by the imperative to self-isolate for extended durations, has potentially resulted in a significant escalation in the tendency to self-diagnose and self-medicate. A study conducted in 2024 found that 38.8% of individuals with orthopaedic conditions currently conduct preliminary research before consulting a physician, and 24.5% try to treat themselves with herbal therapies [5].

ChatGPT, the latest advent in AI technology that was launched in 2022, is built on a generative pre-trained transducer (GPT) architecture. It can be defined as a large language model, or commonly known as a chatbot, that can be widely used for information retrieval in healthcare, including uses related to orthopaedics. Numerous publications in the extant literature address the utilization of chatbots in orthopaedics, with a focus on various facets of these systems, ranging from their role in academic writing processes to their capacity to address common inquiries on specialized subjects [8,13,14,15]. Nevertheless, there remain numerous unanswered questions in the literature concerning the consistency, scope, guidance capabilities, and capacity of these large language models to generate patient-based recommendations [16]. To our knowledge, there are a number of studies in the literature on whether patients will be guided correctly when they use AI-powered chatbots to investigate and attempt to self-treat musculoskeletal problems. In 2023, Gwak et al. stated in their study that ChatGPT can provide generally useful medical information and treatment options to patients unfamiliar with shoulder impingement syndrome. However, they also noted the possibility that the information may contain biased or inappropriate content [17]. In 2024, Ah-yan et al. reported that AI-assisted chatbots for self-management of low back pain provided reliable medical advice and helped patients to self-manage [18]. In contrast, in April 2025, Tabanlı and Demirkıran emphasized the limitations of ChatGPT in addressing psychosocial concerns in low back pain and reported the need for clinician supervision [19]. In 2025, Safran and Yildirim investigated the efficacy of AI-assisted chatbots in musculoskeletal rehabilitation. They concluded that the use of AI-assisted chatbots should remain complementary due to the observed limitations of ChatGPT in consistency, completeness and patient-specific clinical assessment ability [20].

As is evident, there is a lack of consensus in the literature on the self-treatment of musculoskeletal problems by AI-assisted chatbots, with the fact that the majority of studies focus on a single specific disorder being a significant limitation. The objective of this study is to evaluate the effectiveness, reliability, readability, and comprehensibility of the responses provided by an AI-powered chatbot when questioned about the most prevalent orthopaedic problems. Consequently, this study aims to ascertain the occurrence of misguidance and the necessity for an audit of the disseminated information.

## 2. Materials and Methods

ChatGPT 4.0 (ChatGPT Version 4.0; OpenAI, 2024) was presented with 26 open-ended questions, designed to cover the most common pathologies that result in referral to orthopaedic outpatient clinics. The questions were prepared in English by two board-certified orthopaedic surgeons who were actively involved in primary patient care, had participated in survey studies previously, and have native English proficiency. In the preparation of the questions, the most common reasons for referral to orthopaedic outpatient clinics were thoroughly considered, and the content was designed to span a range of levels, from general to specific. The questions were meticulously designed to mirror the standardized user experience and crafted to be asked by the patients themselves (see Table 1 for details). The questions were presented in a single trial to evaluate the immediate response performance of ChatGPT 4.0, and the answers were recorded. The authors state that artificial intelligence was used only at this point in the study process and that no support was received in any other process of the preparation of the manuscript. No ethics committee approval was obtained for this study, in which no patient or personal data was used.

The responses were evaluated at two-month intervals, with intra-rater reliability being assessed (Table 2). The objective of these two distinct evaluations was to demonstrate the consistency and concordance of the authors’ responses. In the evaluation conducted two months later, the questions were not asked again to ChatGPT 4.0; instead, the answers recorded in the first questioning were re-evaluated. This is a precautionary measure to ensure consistency in the evaluation process, particularly in the event of any updates to the AI system during the two-month period. The purpose of this procedure was to ensure that there are no discrepancies in the scores reported by the authors, possibly due to a potential update. The evaluation of the responses was conducted employing Likert scaling. In accordance with this methodology, each author assigned a rating to the questions from 1 to 5, with 1 representing “flawed”, 2 representing “inadequate”, 3 representing “acceptable”, 4 representing “adequate”, and 5 representing “excellent”. The evaluations were conducted in three distinct categories for each question: “diagnosis” (diagnostic accuracy of the response, whether it can identify the targeted diagnosis), “recommendation” (whether self-treatment was recommended and the effectiveness of the self-treatment) and “referral” (whether the chatbot directed the patient to a physician and the accuracy of the referral time). The responses generated by ChatGPT 4.0 were then subjected to a detailed analysis, which involved the categorization of these responses into distinct subgroups. This categorization was based on the specific areas of interest of orthopaedics, namely pediatric, sport, degenerative, infective, and trauma, as well as anatomical regions, including upper extremity, spine, hip, knee, foot & ankle. The “degenerative” subheading was planned to cover deformities secondary to degeneration and the “trauma” subheading was planned to cover complications after trauma surgery.

In addition to the scope of ChatGPT’s responses to patient inquiries, another salient point pertains to the readability and the comprehensibility of the provided answers. As mentioned before, the questions asked of ChatGPT 4.0 were designed to emulate those asked by patients. The readability and the comprehensibility of the responses were evaluated employing the Flesch–Kincaid Reading Ease Score (FRES) and Flesch–Kincaid Grade Level (FKGL). The FRES, ranging from 0 to 100, has been shown to correlate positively with reading ease, with higher values indicating greater ease of reading. The FRES has been demonstrated to serve as a reliable predictor of the level of education required to comprehend the text, otherwise known as the “estimated reading level”. In contrast, the FKGL is an updated version of the FRES, with higher values indicate greater complexity. That is to say, a text with a higher FRES and a lower FKGL score is easier to understand, even for individuals with lower levels of education, and is not complex [21,22,23,24]. Each response in each question was evaluated independently using these scales, and the readability of the response and the level of education required to comprehend the text were recorded.

Statistical analyses were conducted utilizing IBM^®^ SPSS^®^ Statistics Version 26. Mean and standard deviation were employed as descriptive statistics. Cohen’s Kappa analysis was used to assess intra-rater reliability, Kruskal–Wallis analysis was used to ascertain statistical differences between multiple groups and Mann–Whitney U analysis was used for post hoc evaluations. *p* values less than 0.05 were considered significant. The level of agreement was interpreted using kappa values.

## 3. Results

In this study, ChatGPT 4.0 was found to provide consistently high-quality responses across all categories and evaluation criteria, with a majority of the answers being rated as ‘adequate’ or ‘excellent’ (see Table 3). However, the findings of both authors indicate a significant difference in the diagnostic aspect of the responses according to the area of interest (*p* = 0.007 and *p* = 0.001, respectively) (Table 3). Subsequent post hoc analyses revealed that this difference is attributed to the trauma-related questions (Table 4). Subgroup analyses according to anatomical region revealed no other significant differences in the diagnosis, recommendation, and referral aspects of the responses (Table 3).

The mean FKGL score was calculated as 7.8 ± 1.267 (range: 5.8–10.9), and the mean FRES was calculated as 52.68 ± 8.6 (30.2–65). The average estimated reading level required to understand the text was rated as “high school”. In the subgroup analyses according to area of interest and anatomical region, no significant difference was observed between the groups in terms of readability and comprehension of the text (*p* > 0.05), but it was observed that the average estimated reading level required to fully understand the answers was at the “college” level for the questions on infection and the questions related to the knee (Table 5).

## 4. Discussion

The relentless progression of technology, resulting in the affordability and accessibility of information, has led to a growing interest in self-diagnosis and self-treatment due to people’s desire to solve their own problems and their fear of hospitals and infection [6,7,8,9,10,11,12]. The most significant contribution of our study to the existing literature is the validation of the responses of ChatGPT 4.0, the latest advancement in technology, to the most frequently encountered musculoskeletal system disorders in referral to orthopaedic outpatient clinics. Moreover, the evaluation of the responses focused not only on their diagnostic efficiency but also on the validity of the self-treatment suggestions and the timing of referral to a physician. The most striking finding of our study was that although the majority of the responses were found to be “adequate” or “excellent”, the diagnostic effectiveness of the responses to trauma-related questions was found to be weaker (*p* < 0.05). Furthermore, the average educational level required for the readability and the comprehensibility of the responses was found to be “high school”. However, while not reaching statistical significance, it was observed that a “college” level of education was required to comprehend the answers related to the infection-related and knee-related questions.

The literature contains a number of contradictory opinions regarding the reliability of ChatGPT in healthcare. A significant number of studies conducted in 2023 and 2024 investigated the utilization of ChatGPT in the fields of sports medicine, pediatric orthopaedics and hip and knee arthroplasties, yielding favorable outcomes [15,25,26,27,28,29,30,31]. Conversely, Wright et al. documented a solely 59.2% satisfactory response rate for ChatGPT in inquiries concerning total knee and hip arthroplasties in 2024 [32]. Johns et al. investigated the reliability of ChatGPT regarding anterior cruciate ligament reconstruction in 2024 and stated that most of the responses were “outdated” [33]. In 2025, Schwartzman et al. examined the source of information of ChatGPT and reported that there were many repetitions and invalid references [16]. In the present study, it was observed that the diagnostic efficiency (4.77 ± 0.59 and 4.88 ± 0.33, respectively), recommendation capability (4.54 ± 0.86 and 4.62 ± 0.8, respectively), and referral accuracy (4.88 ± 0.83 and 4.96 ± 0.2, respectively) of the responses of ChatGPT 4.0 were considered to be highly satisfactory. Nevertheless, the reliability of ChatGPT’s diagnostic capability in trauma-related questions remains a subject of debate, according to the subgroup analysis conducted within the area of interest (Table 3 and Table 4). The authors of the study reported that the diagnostic efficiency of the chatbot’s responses regarding trauma complications was insufficient (3.75 ± 0.96 and 4.25 ± 0.5, respectively). While the majority of responses were evaluated as “adequate” or “excellent”, the observed differences in diagnosis for trauma-related questions raise concerns about the accuracy of AI-generated chatbots’ knowledge of musculoskeletal health. In investigating the reasons for this situation, the first hypothesis that emerges is the sudden nature of the trauma. The utilization of an AI-powered chatbot, such as ChatGPT 4.0, enables access to pre-existing knowledge found in the extant literature. However, it is important to note that the management of trauma and associated complications does not necessarily follow a textbook approach but is rather patient-based and often involves a combined approach. It is evident that ChatGPT’s capacity to formulate tailored, context-appropriate recommendations is limited, thus necessitating the supervision of a clinician. In the context of online research aimed at self-diagnosis, consultation with expert opinion is imperative, particularly in cases pertaining to trauma-related questions. Nevertheless, the high ratings for self-treatment and referral can be regarded as encouraging indicators that patients are receiving appropriate guidance.

A fundamental aspect of patients undertaking their own research is the importance of comprehensible information. While a substantial amount of evidence-based information is made available in the literature, on physicians’ own websites or on physician education platforms, it is not always possible for patients to properly understand this information, which includes technical terms. Consequently, patients often resort to alternative sources, such as social media or AI-powered chatbots. However, the utilization of plain language and reduced technical terms on these platforms is another topic that requires further research. There have been several reports in the literature on the readability and comprehension of ChatGPT responses. Gül et al. reported that ChatGPT responses to subdural hematomas were complex, difficult to read and required a university degree to understand [24]. Reyhan et al. found ChatGPT responses to questions about keratoconus to be difficult to read and understand [34]. Hancı et al., in their study published in 2024, reported that the readability of ChatGPT responses on palliative care was not sufficient [23]. The present study also set out to evaluate the readability and comprehensibility of the answers provided by ChatGPT 4.0 and to analyze the average level of education required to understand these answers. The mean FKGL score in this study was found to be 7.8 ± 1.267 (range: 5.8–10.9), with a mean FRES of 52.68 ± 8.6 (range: 30.2–65). These results indicate that the responses fell within the accessible range for “high school” graduates. This suggests that ChatGPT 4.0 has the potential to address a broad demographic of patients in terms of musculoskeletal health, though potentially at the expense of nuance and depth in complex cases. However, it is crucial to emphasize that, despite the language being simplified, there is a possibility that crucial clinical details that a healthcare professional would provide may be unintentionally excluded. The contradiction between the results of our study and the literature may be related to the content evaluated. In fact, most of the readability analysis studies in the literature focus on specific conditions (keratoconus, subdural hematoma), whereas our study focused on the most common musculoskeletal disorders. This may have resulted in less technical terms and more understandable responses from the chatbot. In fact, readability becomes more difficult for questions related to the knee and infections, where the situation under investigation becomes more specific.

The significance of artificial intelligence (AI) in healthcare is progressively growing in all areas, including patient education and guidance. In the context of musculoskeletal disorders, where self-diagnosis and self-treatment are prevalent, AI-supported chatbots emerge as a prominent solution. These chatbots offer instantaneous accessibility and rapid response capabilities, rendering them a valuable resource. In this study, we observed that ChatGPT 4.0 provides unbiased, comprehensible, and exhaustive answers in the domains of diagnosis, recommendation, and referral for common musculoskeletal disorders, with no erroneous content and generally satisfactory answers. These answers can be used to support patients’ self-diagnosis and self-treatment tendencies. Conversely, it is imperative to acknowledge that AI and AI-assisted chatbots are subject to perpetual development and refinement. The contents of our work, and of all AI-related works, may become outdated as the system evolves and updates. Subsequent studies may offer additional insights into this matter. Nevertheless, we are confident in the validity and reliability of the results of our study, at least for the present moment. However, it is also important to acknowledge the limitations of this study. Firstly, although a readability analysis was conducted, variables such as education level and occupation may have affected reading skills. The most significant shortcoming of the study is that it was a one-way assessment and readability analysis and did not investigate what patients received from the responses. Furthermore, the analysis was restricted to the 26 most prevalent problems referred to orthopaedic outpatient clinics. While this approach provides a general overview, it would be beneficial to employ more comprehensive and diverse questioning and to analyze the responses in order to evaluate the limitations of the chatbot, which was found to be diagnostically inadequate for trauma-related issues. Additionally, the selection of questions, crafted to encompass a wide demographic of orthopaedic patients, may have contributed to ChatGPT’s favorable performance. As previously documented, ChatGPT is a chatbot that is inadequate for use in specific patient-based situations. A detailed assessment of a specific area rather than a general approach may yield different results. Moreover, while the study constructed patient scenarios based on authors’ real patient-based experiences, the absence of real patient data to make the findings more robust and clinically meaningful is a significant limitation.

## 5. Conclusions

ChatGPT 4.0 is a noteworthy platform that facilitates the self-diagnosis and self-treatment tendencies of patients with musculoskeletal disorders who wish to conduct their own research and access satisfactory answers. However, it is imperative for patients to have a robust understanding of the limitations of chatbot-generated advice, particularly in cases involving musculoskeletal conditions that necessitate the expertise of a professional, such as trauma-related conditions. The findings of this study emphasize the necessity for ongoing enhancement of AI-driven healthcare tools, with a particular emphasis on the improvement of diagnostic accuracy and the assurance of the comprehensibility of medical content.

## Figures and Tables

**Table 1:** Questions asked to ChatGPT 4.0 that reflect the most common orthopaedic problems.

AoI	Anat	Question
Ped	Knee	*My 12-year-old son has been getting more and more pain in the front of his knee when he’s been playing sports and in the evenings. He’s also got a bit of a swelling going on. Any ideas what I should do?*
Ped	F&A	*My 3-year-old daughter’s feet are pressing inwards. What should I do?*
Ped	Hip	*My grandson was just born. I’ve heard about something called a hip ultrasound. Do you know what this is? And when should I have it?*
Ped	Spine	*My 17-year-old daughter has a curve in her back. What should I do?*
Ped	Hip	*My 6-year-old child walks with a limp. Do you have any advice?*
Sport	Up	*My 48-year-old mum has really bad pain in her right shoulder when she gets plates from the top shelves and washes her hair in the shower. Any ideas what she can do?*
Sport	Up	*I’m a 45-year-old housewife and the outer side of my elbow really hurts when I squeeze a cloth or open a jar lid at home. What should I do?*
Sport	Knee	*My mate at work told me that his knee swelled up after he hurt it playing football a month ago. He said it felt like it was rotating and he felt a gap in it. What should he do?*
Sport	F&A	*During the same match, another friend heard a loud pop behind his ankle, which we all heard. But the man can walk. Any ideas what it is?*
Sport	Knee	*I’m 35 and had meniscus surgery last week. Do you think I should get physical therapy?*
Sport	Hip	*My 33-year-old cousin played football when he was younger. He’s been in pain in his left groin for the last three years. Any ideas?*
Sport	Spine	*I am 25 years old and my back hurts. What should I do?*
Deg	Spine	*I am 75 years old and my back hurts. What should I do?*
Deg	F&A	*I’m a 55-year-old woman and I’ve had pain in my heel every morning for the last month. It eases up during the day, but kicks in again in the morning. Any ideas what I should do?*
Deg	Knee	*My dad is 65 and only has pain in his knees when he’s going up and down stairs, but he’s fine when he’s walking on flat surfaces. His knee doesn’t lock, but he does have a bit of pain when going up and down stairs. Any ideas what I should do?*
Deg	Knee	*I’m 75, and my knees hurt a lot when I bend over and stand up. I can’t even walk 100 m Any ideas what I can do?*
Deg	F&A	*My 48-year-old wife has a deformity of the big toe. Do you know what we’re supposed to do?*
Deg	Spine	*I’m a 40-year-old office worker and I’ve had numbness in my left hand and neck for about a month. Any ideas what I should do?*
Inf	Knee	*I’m 26 and got back from Thailand last week. My right knee’s all swollen, warm and really painful. Any ideas what I should do?*
Inf	Knee	*My 60-year-old mum had PRP last week, and now the knee where they did it is swollen and she can’t move it much. Do you know what is it?*
Inf	Hip	*My 6-year-old daughter had the flu last week, and this week she’s got a bit of pain in her hip and is limping a bit. Wat I should do?*
Inf	F&A	*My dad, who’s 75, has had a black, stinky discharge on his foot for about three months. He also can’t feel some parts of his foot. Any ideas what we should do?*
Tra	F&A	*I broke my tibia bone and had to have a nail put in it, and it’s been six months but I’m still in pain. Do you know what I can do?*
Tra	F&A	*I broke my tibia bone and had a nail put in it during surgery. It’s been 10 months and I’m still in pain. Any ideas what I can do?*
Tra	Up	*My 80-year-old grandmother took a tumble at home a week ago, and her left wrist is still all swollen and sore. What we can do?*
Tra	Hip	*I’m 80 and had surgery after a hip fracture six months ago. I start limping when I get tired while walking. Any idea why?*

AoI: Area of interest, Anat: Anatomic Region, Ped: Pediatric, Deg: Degenerative, Inf: Infective, Tra: Traumatic, F&A: Foot and ankle, Up: Upper extremity.

**Table 2:** Analysis of intra-rater reliability.

		Intra-Rater Reliability			
		First Evaluation	Second Evaluation	Kappa	Level of Agreement
**First Author**	**Diagnosis**	4.77 ± 0.587	4.81 ± 0.491	0.859	Strong
	**Recommendation**	4.54 ± 0.859	4.58 ± 0.809	0.912	Almost Perfect
	**Referral**	4.88 ± 0.431	4.88 ± 0.431	1.000	Perfect
**Second Author**	**Diagnosis**	4.88 ± 0.326	4.85 ± 0.464	0.816	Strong
	**Recommendation**	4.62 ± 0.804	4.65 ± 0.797	0.894	Strong
	**Referral**	4.96 ± 0.196	4.96 ± 0.196	1.000	Perfect

Mean ± standard deviation and minimum–maximum range values were used as descriptive statistics.

**Table 3:** Comparison of ChatGPT 4.0 response scores by categories.

		First Author			Second Author		
		Diag	Recom	Referral	Diag	Recom	Referral
**Overall Score**		4.77 ± 0.59 (3–5)	4.54 ± 0.86 (2–5)	4.88 ± 0.43 (3–5)	4.88 ± 0.33 (4–5)	4.62 ± 0.8 (2–5)	4.96 ± 0.2 (4–5)
**AoI**	**Ped** (*n*= 5)	4.8 ± 0.45 (4–5)	4.2 ± 0.84 (3–5)	5 (5)	5 (5)	4.4 ± 0.89 (3–5)	5 (5)
	**Sport** (*n* = 7)	5 (5)	4.71 ± 0.77 (3–5)	4.57 ± 0.79 (3–5)	5 (5)	4.86 ± 0.38 (4–5)	5 (5)
	**Deg** (*n* = 6)	5 (5)	4 ± 1.27 (2–5)	5 (5)	5 (5)	4 ± 1.27 (2–5)	4.83 ± 0.41 (4–5)
	**Inf** (*n* = 4)	5 (5)	5 (5)	5 (5)	5 (5)	5 (5)	5 (5)
	**Tra** (*n* = 4)	3.75 ± 0.96 (3–5)	5 (5)	5 (5)	4.25 ± 0.5 (4–5)	5 (5)	5 (5)
	***p***	*0.007*	0.136	0.227	*0.001*	0.189	0.504
**Anat**	**Up** (*n* = 3)	5 (5)	4.33 ± 1.16 (3–5)	4.33 ± 1.16 (3–5)	5 (5)	4.67 ± 0.58 (4–5)	5 (5)
	**Spine** (*n* = 4)	5 (5)	4.75 ± 0.5 (4–5)	5 (5)	5 (5)	5 (5)	5 (5)
	**Hip** (*n* = 5)	4.6 ± 0.89 (3–5)	4.8 ± 0.45 (4–5)	5 (5)	4.8 ± 0.48 (4–5)	4.6 ± 0.89 (3–5)	5 (5)
	**Knee** (*n* = 7)	5 (5)	4.43 ± 1.13 (2–5)	5 (5)	5 (5)	4.43 ± 1.13 (2–5)	4.86 ± 0.38 (4–5)
	**F&A** (*n* = 7)	4.43 ± 0.79 (3–5)	4.43 ± 0.98 (3–5)	5 (5)	4.71 ± 0.49 (4–5)	4.57 ± 0.79 (3–5)	5 (5)
	***p***	0.189	0.974	0.260	0.405	0.834	0.607

Diag: Diagnostic, Recom: Recommendation, Deg: Degenerative, AoI: Area of interest, Anat: Anatomic Region, Ped: Pediatric, Deg: Degenerative, Inf: Infective, Tra: Traumatic, F&A: Foot and ankle, Up: Upper extremity, *p*: Statistical significance value. Mean ± standard deviation and minimum–maximum range values were used as descriptive statistics. Kruskal–Wallis Test was applied for statistical comparison of multiple subgroups. Italicized and underlined values denote statistically significant differences (*p* < 0.05).

**Table 4:** Post hoc analysis of the diagnostic evaluation of the answers of ChatGPT 4.0.

	First Author				
	Pediatric	Sport	Degenerative	Infective	Trauma
**Pediatric**	N/A	0.237	0.273	0.371	0.078
**Sport**	0.237	N/A	1.000	1.000	*0.011*
**Degenerative**	0.273	1.000	N/A	1.000	*0.018*
**Infective**	0.371	1.000	1.000	N/A	*0.046*
**Trauma**	0.078	*0.011*	*0.018*	*0.046*	N/A
	**Second Author**				
	**Pediatric**	**Sport**	**Degenerative**	**Infective**	**Trauma**
**Pediatric**	N/A	1.000	1.000	1.000	*0.025*
**Sport**	1.000	N/A	1.000	1.000	*0.010*
**Degenerative**	1.000	1.000	N/A	1.000	*0.016*
**Infective**	1.000	1.000	1.000	N/A	*0.040*
**Trauma**	*0.025*	*0.010*	*0.016*	*0.040*	N/A

N/A: non-applicable. Mann–Whitney U Test was used for post hoc analysis of the differences found to be significant in the Kruskal–Wallis Test. The values given in the table are *p* values. Italicized and underlined values denote statistically significant differences (*p* < 0.05).

**Table 5:** Readability analysis of the responses of ChatGPT 4.0.

		Flesch–Kincaid Grade Level	Flesch Reading Ease Score	Reading Grade Level
**Overall Score**		7.8 ± 1.267 (5.8–10.9)	52.68 ± 8.6 (30.2–65)	High School
**AoI**	**Ped**	8.26 ± 1.22 (7–10)	50.96 ± 5.75 (43.2–56.4)	High School
	**Sport**	7.93 ± 1.81 (5.8–10.9)	51 ± 12.84 (30.2–65)	High School
	**Deg**	7.35 ± 0.9 (5.9–8.6)	56.4 ± 6.11 (46.9–64.6)	High School
	**Inf**	8.23 ± 0.73 (7.2–8.9)	49.13 ± 4.49 (46.1–55.8)	College
	**Tra**	7.28 ± 1.25 (6.1–8.5)	55.73 ± 9.56 (47–64.2)	High School
	***p***	0.610	0.596	N/A
**Anat**	**Up**	7.3 ± 1.25 (6.3–8.7)	55.73 ± 8.59 (46.7–63.8)	High School
	**Spine**	6.83 ±1.26 (5.8–8.5)	58.2 ± 8.5 (47–65)	High School
	**Hip**	7.96 ± 1.87 (6.1–10.9)	51.76 ± 12.82 (30.2–64.2)	High School
	**Knee**	8.51 ± 0.95 (7.4–10)	48.63 ± 6.27 (39.6–55.3)	College
	**F&A**	7.76 ± 0.87 (6.3–8.6)	52.91 ± 7.32 (46.9–63.8)	High School
	***p***	0.202	0.223	N/A

Deg: Degenerative, AoI: Area of interest, Anat: Anatomic Region, Ped: Pediatric, Deg: Degenerative, Inf: Infective, Tra: Traumatic, F&A: Foot and ankle, Up: Upper extremity, *p*: Statistical significance value. N/A: Non applicable. Mean ± standard deviation and minimum–maximum range values were used as descriptive statistics. Kruskal–Wallis Test was applied for statistical comparison of multiple subgroups.

## Data Availability

The datasets generated during and/or analyzed during the current study are not publicly available but are available from the corresponding author on reasonable request.

## Abbreviations

The following abbreviations are used in this manuscript:

AI	Artificial Intelligence
AoI	Area of Interest
ChatGPT	Chat Generative Pre-trained Transformer
Deg	Degenerative
F&A	Foot and Ankle
FKGL	Flesch–Kincaid Grade Level
FRES	Flesch–Kincaid Reading Ease Score
Inf	Infective
Ped	Pediatric
PRP	Platelet-Rich Plasma
SPSS	Statistical Package for the Social Sciences
Tra	Trauma
Up	Upper Extremity
